# Assessment of infrastructure, behaviours, and user satisfaction of guardian waiting shelters for secondary level hospitals in southern Malawi

**DOI:** 10.1371/journal.pgph.0002642

**Published:** 2024-07-24

**Authors:** Mindy Panulo, Jennifer Lamb, Kondwani Chidziwisano, Blessings White, Robert Dreibelbis, Tracy Morse

**Affiliations:** 1 Centre for Water, Sanitation, Health and Appropriate Technology Development (WASHTED), Malawi University of Business and Applied Sciences, Blantyre, Malawi; 2 Department of Civil and Environmental Engineering, University of Strathclyde, Glasgow, Scotland; 3 Department of Disease Control, London School of Hygiene and Tropical Medicine, London, United Kingdom; 4 Department of Public and Environmental Health, Malawi University of Business and Applied Sciences, Blantyre, Malawi; Jawaharlal Nehru Medical College, INDIA

## Abstract

Guardian Waiting Shelters (GWSs) are an essential component of the Malawi’s health care system as they serve as a temporary home for patient guardians while taking care of their relatives admitted to the hospital, and expectant mothers. Although GWSs play a crucial role in Malawi’s healthcare system, past studies have primarily concentrated on maternity waiting homes, neglecting the role and importance of GWSs. The study examines GWS management structures and conditions, as well as guardian satisfaction and perception of health risks related to GWS use. In this explanatory sequential mixed methods design, we assessed 12 GWSs from southern region of Malawi. Qualitative data included interviews (n = 149) and focus group discussions with patient guardians (n = 72), interviews with GWS caretakers (n = 5), representatives from Hospital Management (n = 12) and Hospital Advisory Committees (n = 11). Lack of guidelines and standards for GWSs resulted in creating a customized facility checklist to quantitatively assess infrastructure present at GWSs (n = 12). Descriptive statistics and qualitative thematic analysis were utilized for data analysis, and a problem tree analysis was used to triangulate and summarize the findings. A total of 249 participants participated in the study. Each GWS had an average of 100 users daily, primarily adult females (71%). No one was accountable for GWS operation and maintenance due to the lack of a management hierarchy. GWS infrastructure conditions were poor, with inadequate functional sleeping rooms, insufficient access to water, sanitation and hygiene facilities. Notably, 50% of the GWSs lacked water access, and a quarter had non-functional toilets. Guardians felt unsafe and at risk of disease transmission when staying within GWS. Study findings highlight lack of clear, consistent GWS ownership as a root cause of challenges in GWSs. Clear policy and operational standards must be established for effective management and smooth functioning of GWSs.

## Background

Sustainable Development Goal (SDG) 3.8 aims to achieve universal health coverage [1]. However, Low- and Middle-Income Countries (LMICs) are struggling to achieve SDG 3 due to a shortage of healthcare personnel, supplies and budget [2–5]. As a result, hospitals in the sub-Saharan Africa region, including Malawi, rely on family members as patient’s carers (locally known as patient guardians) as a key component of health services.

In Malawi, patient guardians are typically accommodated in a dormitory type of housing known as the Guardian Waiting Shelters (GWSs) [6–8]. GWSs are today an integral component of the health care system in Malawi, established to provide a safe and healthy environment for patient guardians to reside in while they attend to their relatives admitted to hospital. Duties of these guardians at hospitals typically involve activities such as bathing, cooking, feeding, monitoring of medications, and assisting with rehabilitation exercises for patients [2,7,9]. Additionally, the physical presence of a family member during hospitalization has shown to offer psychosocial support to the patient, which ensures trust in the care the patient is receiving [10]. In some circumstances, GWSs also serve as Maternity Waiting Homes (MWHs) for women with high-risk pregnancies or those from hard-to-reach areas as they await their expected date of delivery [10–13]. The World Health Organisation (WHO) recommends provision of MWHs in healthcare settings to promote maternal and child health (MCH) [14]. However, in Malawi, specifically designated MWHs have been reported in only 50% of the district hospitals [7].

Despite GWSs providing residence to patient guardians and maternity waiting mothers, previous research has focused on MWHs only, through the examination of their conditions, usage, quality of care, users experience and satisfaction [11,15–19]. Evaluation studies in Malawi, Ethiopia and Zambia found that MWH users were dissatisfied with the sleeping and cooking space, availability of water, lack of privacy, poor sanitation, pests and congestion [11,17,20,21]. Based on findings from a multi country qualitative meta-synthesis, Penn-Kekana and colleagues argue that clean and safe environments are central to the success of MWH [22]. To date, research in Malawi has only evaluated the status of the MWHs model as an integral component of the healthcare service delivery system and not the GWSs model [16–18,23].

This study is part of the larger package of work exploring hygiene conditions in healthcare facilities and GWS in secondary hospitals of Southern Malawi. This specific study presents the results of the initial scoping of GWS across the region which aimed to: 1) assess available infrastructure, 2) understand guardian satisfaction with the GWS structure and environment, and 3) assess guardian’s perception of health risk from GWS use, 4) examine the GWS management structures. Results from this study will help inform service providers, local planners, and decision makers to improve the functionality of GWSs.

## Methodology

### Study design and setting

**Fig 1:**
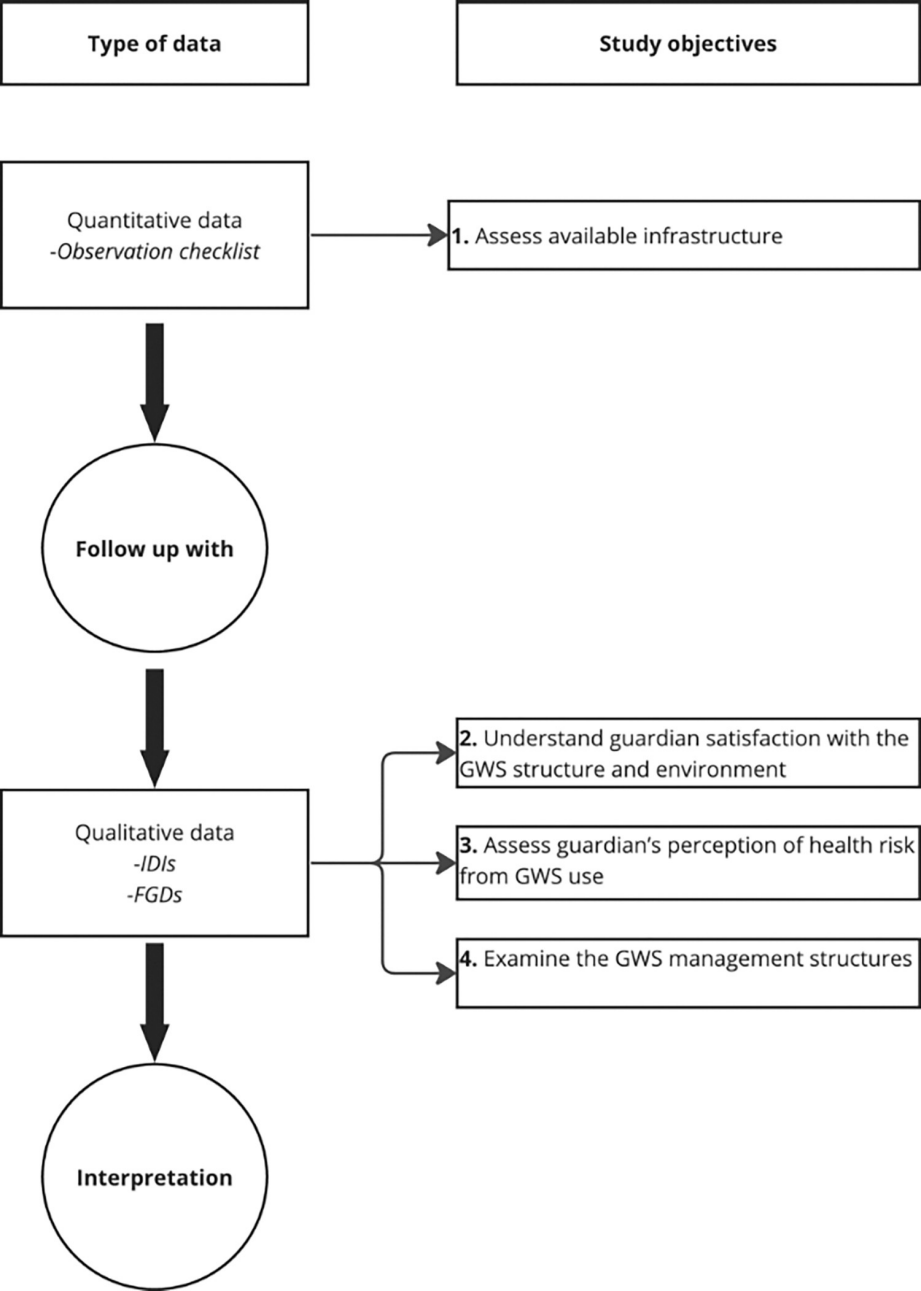
Study design.

This was an explanatory sequential design [24] where the initial phase (quantitative) was followed by qualitative. The study used quantitative data to address objective 1, which involved assessing the infrastructure in the GWS. Qualitative data, on the other hand, addressed objectives 2 to 4 (Fig 1).

The study was conducted in twelve GWSs located across the twelve districts in the southern region of Malawi. These hospitals belong to the second tier of Malawi’s healthcare system where they serve as referral centres for primary healthcare facilities. The hospitals offer outpatient and inpatient services, surgical procedures such as caesarean sections, and other emergency life-saving surgeries. The population which each of these facilities serve varies from 130, 949 to 1,251,484 people with annual admissions ranging from 2, 943 to 18, 220 patients.

### Study population

We recruited multiple population groups for our study. First, we recruited patient guardians, a family member or designated carer of a patient seeking health care, who were currently using the GWS. We also recruited members of the Hospital Advisory Committee (HAC), community representatives who, on voluntary basis, act as mediators between the community members and healthcare workers. The HAC also ensure that there is accountability of medicine at the hospital, the hospital grounds and services are respected and functional, and deliver hygiene talks to patient guardians. Other study participants included members of the hospital management committee, specifically hospital managers tasked with the daily management of the hospital, and a GWS caretaker where present. The caretaker was paid by the hospital and his or her roles included sweeping the public yard area of the GWS, cleaning toilets, solid waste management, ensuring there was water onsite at the GWS and reminding patient guardians to take care of the sleeping, cooking areas and water, sanitation, and hygiene (WASH) infrastructure.

### Sampling and sample size

**Fig 2:**
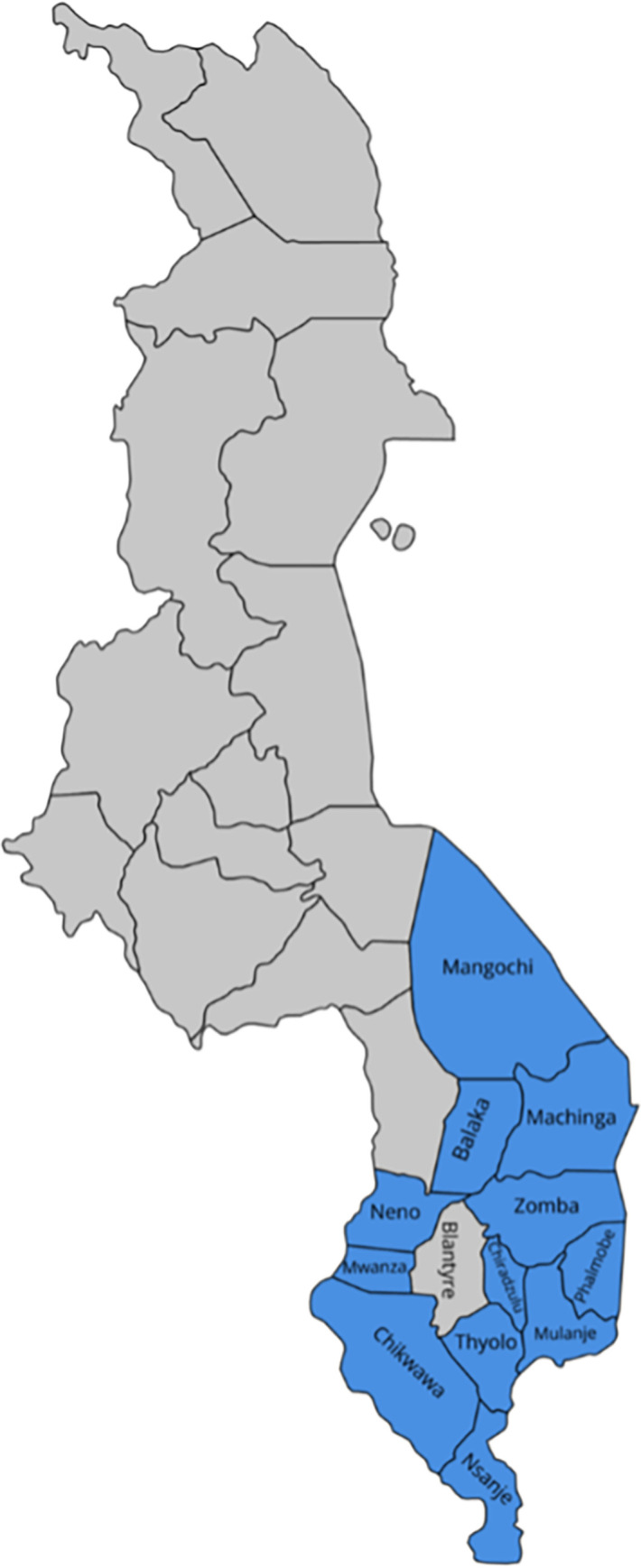
Map of Malawi depicting the districts of the southern region included in the study (Blantyre not included in the sample as it does not have secondary level hospital facility and was therefore excluded).

The study targeted 12 hospitals in the southern region of Malawi that act as referral centres for primary care facilities (i.e., Health centres). Of these 12, ten were district (public) hospitals and two were Christian Health Association (CHAM) (private) hospitals in the southern region of Malawi (Fig 2).

From each hospital, we recruited 1 HAC member, 1 GWS caretaker (if available), 1 Hospital Management Committee (HMC) member and six patient guardians for in-depth interviews (IDIs). Two focus group discussions (FGDs) with patient guardians were also conducted at each hospital (1 male; 1 female) to fully understand the opportunities and challenges surrounding GWSs. Inclusion criteria for all patient guardians included 1) age 18 years or older, and 2) had used the GWS for more than four days to ensure they were familiar with the hospital and GWS setting.

As HAC members act as a bridge between the community and the hospital, we engaged them to understand their involvement in the management of the GWS. We used purposive and convenience sampling. Thus, any HAC member who was available at the time of data collection and had been in the post for more than a year was engaged in the study. Additionally, any Hospital Management Committee member who was present during data collection was engaged. We conveniently sampled and engaged the available GWS caretakers, as in cases where a GWS had a caretaker, it was just one person.

All study participants gave written consent to participate in the study.

### Data collection

Data was collected from October to December 2021 by three experienced research assistants who were fluent with the local language (*Chichewa)*. Two research assistants had Master of Science degrees (MSc Social Anthropology of Development and MSc Environmental Health Sciences) and one research assistant had Bachelor of Science degree in Environmental Health Sciences. These research assistants had experience in qualitative and quantitative data collection and were trained for four days including pre-testing of the data collection tools prior to all of them collecting data. The study applied various methods of data collection to ensure a comprehensive understanding of each GWS setting:

#### Observation checklist

To assess the infrastructure availability, access, quality and functionality we developed an observation checklist. No formal guidelines or standard for GWS in Malawi exist, and a bespoke facility checklist was developed based on assessment tools previously used to assess institutions including Health Care Facilities (HCF). These tools included Service Provision Assessment (SPA) tool, SARA tool, Service Delivery Indicators (SDI) tool, Emory tool, WHO and UNICEF Joint Monitoring Program (JMP) monitoring tool and WASH for Health Facility Improvement Tool [25–29]. The checklist assessed WASH conditions, including access to safe water (water points), hygiene (i.e., presence and cleanliness of the bathing areas, laundry areas, cooking places and presence of handwashing facilities), and sanitary facilities (toilets). Additionally, we assessed availability and condition of infrastructure at the GWS, such as sleeping rooms and cooking spaces. Observations provided insights for further exploration during the subsequent interviews and focus group discussions. Attached checklist in the supplementary information provides details of the various components and how they were assessed.

#### Qualitative data

One hundred In-depth interviews (IDIs) were conducted in total. Six IDIs with patient guardians from each facility (n = 72) were conducted to understand their daily life at the GWS, including hygiene practices, access to water and sanitary facilities, perception of safety and disease risk while staying at the GWS. IDIs with HMC (n = 11) and HAC representatives (n = 12) provided an understanding of the ownership, governance structures, and daily running of the GWS. IDIs with GWS caretakers (n = 5) generated information on daily management of the GWS. The average number of participants per Focus Group Discussion (FGD) with patient guardians was seven. Twenty-one FGDs were conducted to understand social and community regulated practices in each based upon shared common values and beliefs which could not be explored using IDIs. IDIs lasted 45 to 60 minutes while FGDs were conducted for 60 to 90 minutes in the local language.

### Data analysis

#### Quantitative data

Quantitative data from checklist observations were collected through Kobo Collect software platform[30] and later exported to Microsoft Excel 2016 (Microsoft Corporation, Redmond, WA, USA). Descriptive statistics were used to summarize the collected data and categorical variables were summarized using frequencies.

#### Qualitative data

All interviews (IDI and FGD) were audio recorded, and transcripts were manually generated from the recordings. Three research assistants conducted a thematic analysis of all transcriptions. The analysis included the team becoming acquainted with the data and categorizing the data based on predetermined codes. These codes included GWS infrastructure, condition and usage; Guardian’s experience using GWS; perception of public health risk and GWS ownership and management. Researchers coded all transcripts after reaching consensus on final codes. The data was then systematically arranged and documented manually. Quotations, codes, and in-depth analysis of these quotations were recorded in an excel spreadsheet.

### Problem tree analysis

Based on the hypothesis that although patient guardians are integral, they are not supported within the current GWS system, the research team triangulated the thematic areas from the qualitative analysis alongside the quantitative data from the facility checklists using a problem tree analysis. Appropriate for consolidation of complex problems, the problem tree analysis was used to map out the anatomy of the cause-and-effect relationships seen at the GWS [31]. This provided a structured approach to align findings with cause or effect, and frame the challenges related to GWS in a manner which could be effectively communicated to the relevant stakeholders for these facilities and used to support future intervention development.

### Ethical consideration

This study was approved by the National Health Science Research Committee of Malawi (P No. 21/11/2822) and London School of Hygiene and Tropical Medicine Ethics Committee, UK (Ref 2655). All interviewed respondents provided written informed consent for participation in the study, and no names were mentioned or recorded during the FGDs and interviews.

## Results

### Socio-demographic characteristics of respondents

In total, 249 respondents were involved in the study, with 221 participants being patient guardians. Seventy-two patient guardians were interviewed while 149 patient guardians were engaged in FGDs. Forty-two percent (n = 5) GWSs had caretakers who all participated in the study (Table 1). Eleven management committee members and 12 HAC members participated through IDIs.

**Table 1:** Study participants.

GWS name	Affiliation	HAC member (IDI)	Hospital management committee member (IDI)	GWS caretaker (IDI)	Patient guardians		
					Number per FGD		IDIs
					Male FGDs	Female FGDs	
Chiradzulu District Hospital GWS	Public HCF	1	1	-	-	6	6
Thyolo District Hospital GWS	Public HCF	1	1	1	7	7	6
Mulanje District Hospital GWS	Public HCF	1	1	1	-	7	6
Zomba St. Lukes GWS	Private HCF	1	-	1	-	9	6
Machinga District Hospital GWS	Public HCF	1	1	-	8	8	6
Mangochi District Hospital GWS	Public HCF	1	1	1	7	7	6
Chikwawa District Hospital GWS	Public HCF	1	1	-	7	7	6
Nsanje District Hospital GWS	Public HCF	1	1	-	6	6	6
Balaka District Hospital GWS	Public HCF	1	1	-	7	7	6
Mwanza District Hospital GWS	Public HCF	1	1	-	8	8	6
Phalombe Holy Family GWS	Private HCF	1	1	1	6	7	6
Neno District Hospital GWS	Public HCF	1	1	-	7	7	6
**Total**		12	11	5	149		72

Across all 221 guardians who participated in the study, their ages ranged from 19 to 60 years old, with a median of 43 years old. Forty-three percent (n = 96) of these guardians were supporting antenatal waiting mothers (S1 Appendix). Seventy-nine percent of the patient guardians depended on public transport to get to the hospital/GWS and the duration of stay within the GWS varied between 5 to 55 days, with a median stay of 22 days.

The study findings have been organized and presented according to the specified themes and sub-themes that were generated based on the study objectives as summarized in Table 2.

**Table 2:** Predetermined study themes and sub-themes.

Objective	Theme	Sub themes
Assessing available infrastructure	GWS infrastructure	• Available infrastructure • GWS infrastructure condition • GWS infrastructure usage
Understanding guardian satisfaction with the GWS structure and environment	Guardian’s experience using GWS	• WASH behaviours of guardians • Social relationships • Accountability
Assessing guardian’s perception of health risk from GWS use	Guardian’s perception of public health risk	• Contracting and transmitting diseases • Well-being concerns i.e., safety, security and privacy
Examine the GWS management structures	GWS ownership and management	• GWS ownership • GWS budget • GWS management

### Guardian waiting shelters infrastructure, condition and usage

During the time of the study, an average of 100 people were reported to be utilising each GWS daily, with a range of 114. Forty-two percent of the facilities (n = 5) had no specific accommodation for the maternity waiting mothers (Maternity Waiting Shelters), in such case, GWSs were used to also accommodate both maternity waiting mothers and patient guardians.

**Fig 3:**
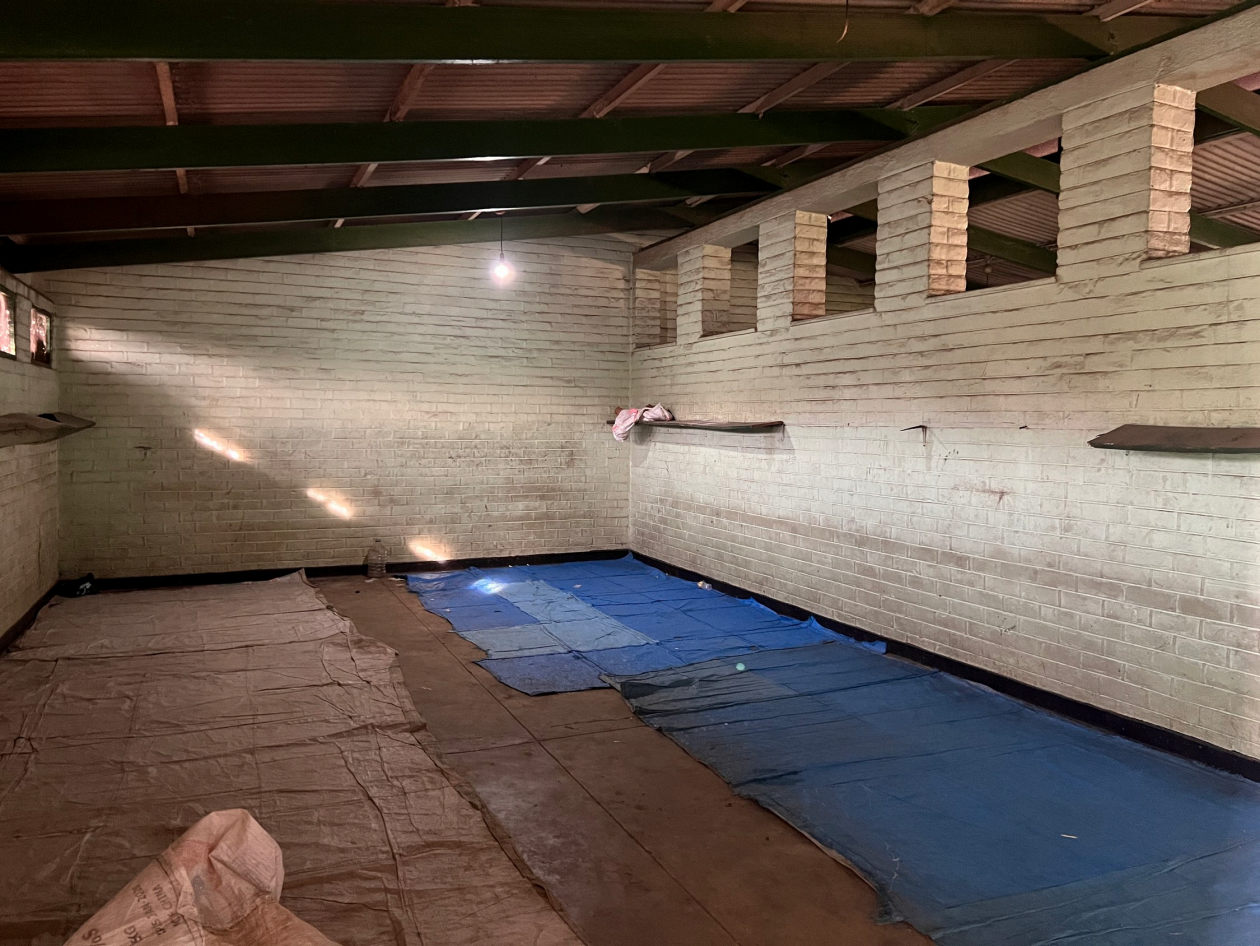
Standard sleeping room for GWS.

Fifty-eight percent (n = 7) of the GWSs had sleeping rooms used by the patient guardians (Table 3) (Fig 3). In the remaining 43% (n = 5) of GWSs, three were not used by the patient guardians due to poor conditions (i.e., no doors, windows and partly roofed), while two GWS had no sleeping rooms. In these cases, the patient guardians reported sleeping in patient wards, hospital verandas and corridors (Table 3). None of the GWSs had a supply of basic amenities or security features, such as mosquito nets, bedding, lockable sleeping room doors or a secure place to store their belongings. Eighty-three percent (n = 10) of GWSs, all serving public facilities, lacked electric lighting (Table 2). GWS serving private owned healthcare facilities had better amenities such as electricity, security guards, and perimeter fencing around the GWS (Table 3).

**Table 3:** GWS Characteristics for twelve southern region health facilities.

Facility name	District Population	Ownership		Management		Estimated number of guardians	Usage of GWS (Sleeping and or cooking)	Location of GWS	Security			Availability of water source within the GWS	Toilets			Bathrooms			Presence of handwash facilities	GWS Payment of user fee
		Public/private facility	Responsibility for GWS	Presence of caretaker	Presence of chairperson				Presence of guard	Fenced	Electricity		Presence of toilet within the GWS	Number of toilets	Condition of the toilets	Presence	Number of bathing shelters	Functionality of the bathing shelters		
Chiradzulu District Hospital GWS	356,875	Public	District Council	´	´	70	cooking only	outside hospital	´	´	´	´	√	4	all latrines are full, floor not clean, presence of faeces on latrine walls, bad smell	√	4	all functional	´	´
Thyolo District Hospital GWS	721,456	Public	District Council through the Hospital	´	√	100	cooking only	outside hospital	´	´	´	√	√	6	1 collapsed, 2 are full. The filled toilets are still being used plus the other 3, there is presence of faeces on latrine walls,	√	8	all Functioning	´	´
Mulanje District Hospital GWS	684,107	Public	Community through HAC	√	√	112	sleeping + cooking	outside hospital	´	´	No. However, wiring seen)	√	√	4	all latrines are full, presence of solids in the toilet, presence of faeces in latrine walls	√	4	Only 1 in use	´	√
Zomba St. Lukes GWS	851,737	Private	Hospital	√	√	56	sleeping + cooking	within hospital premise	´	√	√	√	√	6	4 in good condition, 2 closed	√	4	All functional	√	´
Machinga District Hospital GWS	735,438	Public	District Council through the Hospital	´	√	130	sleeping + cooking	within hospital premise	´	√	√	√	√	10	all latrines are full, floor not clean, presence of faeces in latrine walls	´	0	NA	´	√
Mangochi District Hospital GWS (General)	1,148,611	Public	Hospital	√	´	120	cooking only	within hospital premise		√	´	´	√	8	8 permanent pit latrines + 7 COVID-19 emergency latrines (2 of which are still in use, given the poor conditions of the permanent facilities	√	2	Not functional—locked	´	´
Chikwawa District Hospital GWS	564,684	Public	Hospital	´	´	95	cooking only	within hospital premise	´	√	´	´	√	2	´	√	8	all functional	´	´
Nsanje District Hospital GWS	299,168	Public	Community through HAC and District Council	´	√	70	sleeping + cooking	outside hospital	´	´	´	´	√	3	the toilets are not clean, all toilets are filled up.	√	3	Two are in use, one is not in usable condition	´	´
Balaka	438,379	Public	Community through HAC and the Hospital	´	√	110	Sleeping + cooking	within hospital premise	´	√	´	√	√	2	All toilets are filled up, and not clean	√	2	all functional	´	Yes
Mwanza	130,949	Public	District Council	´	´	60	cooking only	outside hospital	´	´	´	´	´	´	NA	NA	NA	NA	NA	´
Phalombe	429,450	Private	Hospital	√	√	100	sleeping + cooking	outside hospital	√	√	√	√	√	4	the toilets are clean, but there is water on the floor	√	5	all functional	√	√
Neno	138,291	Public	Hospital and District Council	´	√	130	sleeping + cooking	outside hospital	´	√	´	´	√	2	filled up and not clean	√	4	3 are in use, one not in use	´	´

Note: √ = Present at the time of data collection ´ = Not present at the time of data collection.

**Fig 4:**
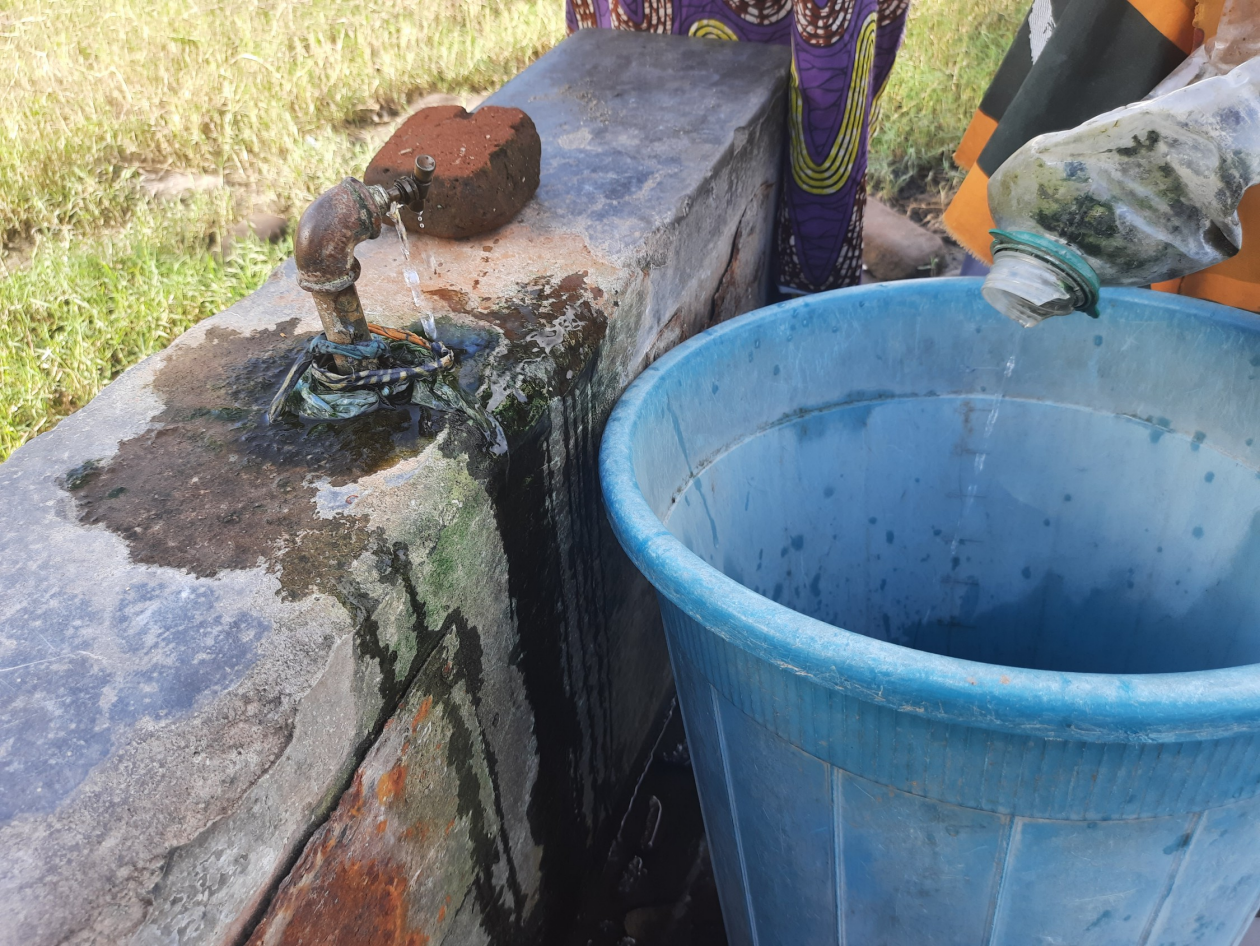
Broken water tap located within the Guardian waiting shelter.

Fifty percent (n = 6) of the GWSs had a functional onsite water supply from a safe source (standpipes and boreholes) within the GWS, while others accessed boreholes or standpipes from community, school and hospital sources (Table 3). Missing or broken taps were commonly observed at GWSs (Fig 4). Respondents reported lack of timely and correct payment of pre-paid water, pumps being broken and sporadic electricity limiting water availability. Ninety-two (n = 11) of the GWSs had toilets (Table 3). The two GWSs serving private owned healthcare facilities which had both water closet toilets and pit latrines. The remaining nine GWSs with toilets that serve public healthcare facilities had only pit latrines, of which seven were almost full. Used nappies, faeces and menstrual hygiene materials were observed on the floor (Table 3). Twenty-five percent (n = 3) of GWSs had evidence of open defaecation.

**Fig 5:**
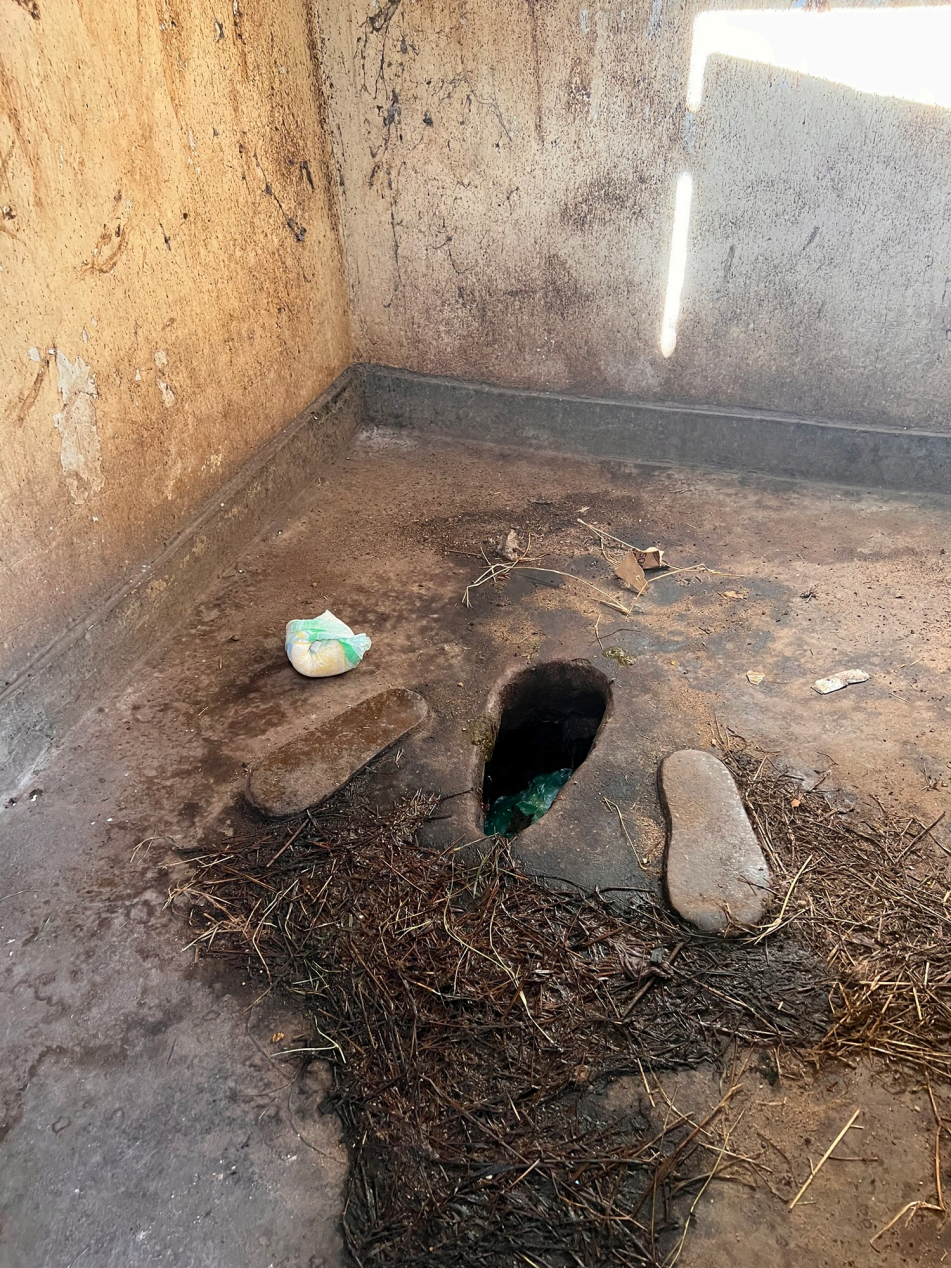
Guardian waiting shelter pit latrine, which is full, with indiscriminate disposal of diapers.

**Fig 6:**
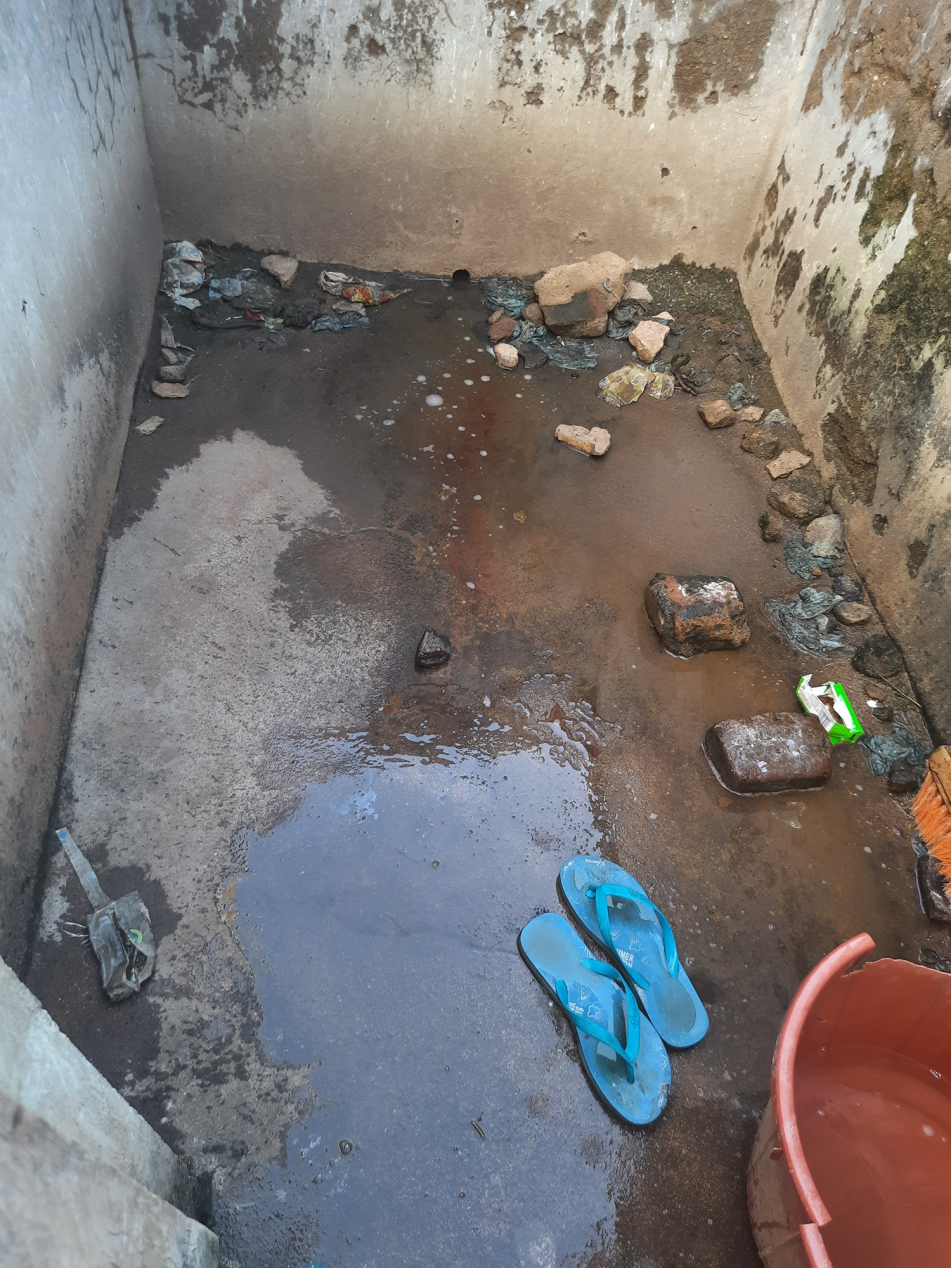
Guardian waiting shelter bathing shelter used by both male and female patient guardians.

Hygiene conditions were generally better in GWSs at private owned healthcare facilities compared to those serving public healthcare facilities. Handwashing facilities (manually filled 20-litre buckets with taps) were observed only in the GWSs at private owned healthcare facilities (Table 3). However, soap (liquid or bar) was not observed at any handwashing point. The majority of GWSs (n = 11) had adequate facilities for guardians to bathe (bathrooms). However, GWSs at public healthcare facilities were observed to be in poor condition (i.e., poor drainage, no privacy and were left uncleaned) (Figs 5 and 6). Due to unhygienic conditions of the toilets, guardians reported feeling disgusted by latrine conditions and resorted to using bathing areas as toilets and or opted for open defaecation. Only one GWS had information education and communication (IEC) materials (poster) displayed within the GWS to promote handwashing with soap.

Forty-two percent (n = 5) of the GWSs had rubbish pits which were found to be full of waste from the GWS, and often located close to the cooking areas. Market sellers nearby the GWSs were also reported to use the GWS infrastructure–specifically toilets and rubbish pits. Additionally, people with developmental disabilities were also reported and observed staying in five of the GWS, including using the sleeping and cooking places.

Sixty-seven percent (n = 8) of the GWS reported offering health and hygiene talks to the patient guardians either on a daily, weekly, or ad-hoc basis (e.g., during outbreaks). These talks were conducted by the GWS caretaker, guards, HAC members, or health personnel, and were confirmed by the patient guardians. Talks contained various messages covering sanitation, such as solid waste management or use of latrines; hand hygiene and potential risks and health concerns of diarrhoea, cholera, COVID-19, scabies, malaria, cervical cancer, Tuberculosis, and HIV.

### Guardian’s experience using Guardian waiting shelters

#### Water, sanitation, and hygiene (WASH)

All patient guardians appreciated the presence of the GWS within the hospital and reported using the GWS as their home as it offered them a place to cook, bathe, and for some, to sleep. At better maintained facilities, guardians reported being able to practice selected hygiene behaviours while at the GWS such as bathing, sweeping and mopping sleeping and cooking areas, hand washing before eating, washing clothes, washing kitchen utensils, washing food before cooking, or covering food and water, and disposing or reheating left-over food. Motivation to practise such behaviours were reported as preventing disease, a continuation of what they practised at home, and having more free time while at the GWS. Additionally, some patient guardians reported that since they found the GWS already clean, they were motivated to ensure they maintained these standards.

However, where infrastructure was limited or poor, patient guardians reported poor hygiene practices. For example, due to lack of laundry spaces, patient guardians washed soiled clothing including bedding at drinking water collection points, which discouraged others from using such water points and resulted in them collecting water from alternative sources located outside the GWS premise. It was further reported that female patient guardians were leaving menstrual hygiene materials in the bathing shelters, as they had no appropriate means for disposal. Since most of the GWS were utilised by women, shelters for bathing were not segregated by gender and one male IDI participant commented:

“Sometimes I fail to bathe here at the GWS because it is shameful for me to find menstrual hygiene materials in the bathrooms” (IDI, Male guardian).

#### Cooking and food hygiene

Cooking meals for patients was considered one of the primary responsibilities of the patient guardians. Guardians cooked from various locations within the GWS, e.g. on the ground of the GWS compound area, in old sleeping rooms or in designated kitchens. Food preparation was generally considered challenging by all guardians. Guardians reported that the cooking rooms were inadequate and often lacked ventilation, especially considering that main source of fuel was biomass. The lack of handwashing facilities in the cooking spaces was reported to lead to inadequate or no handwashing during cooking. Limited kitchen utensils availability (e.g., dish racks and plates) was further reported to compromise food hygiene practices. Patient guardians felt that the lack of WASH facilities compromised their ability to practice food hygiene which may subsequently affect both guardians and patients.

“Due to lack of proper cooking place, we cook in those old sleeping rooms which are small and poorly ventilated. We fear this may cause cough and other respiratory diseases” (Female FGD participant).

#### Social relationships

Guardians were very dependent on financial support during the time they spent at the facility–usually receiving money, food and firewood from friends and neighbours. Many reported that financial support was insufficient, leading them to seek piece work in the surrounding area, often resulting in tensions with local vendors or labourers. Additionally, patient guardians also reported fetching and selling firewood to other patient guardians at GWS to have money. Quality of the relationships between patient guardians varied across the GWSs. In some instances, patient guardians indicated good relationships in terms of sharing food and firewood and escorting one another during the night from the GWS to the hospital. On the other hand, some patient guardians complained of theft between patient guardians, taking items such as cooking utensils, clothes, and food.

“I remember I came to the hospital unplanned as my patient had an emergency condition. But when I arrived here at the GWS, my fellow patient guardians gave me food for my patient” (IDI, Male guardian).

#### Accountability

Channels by which patient guardians could engage with those in charge of the GWSs were unclear. This was compounded in many cases by the lack of clarity, even at management level, of where responsibility for this facility lay. Even when reporting mechanisms were clear, patient guardians reported that they were not comfortable reporting challenges, as they felt doing so would compromise the quality of care given to their patients. In some instances where patient guardians were nominated as the GWS chairperson there was also a belief that the associated patient would not get better and not be discharged within a short period of time. Such a myth therefore potentially discouraged patient guardians volunteering for such a role when staying at the GWS.

### Guardian’s perceptions of public health risk

Patient guardians perceived that the poor environmental health conditions at the GWS, such as overfilled waste disposal sites, poor ventilation, limited access to safe water, sanitation, and hygiene (WASH) infrastructure and poor cleaning standards, exposed them to specific health risks. Specifically, the patient guardians felt vulnerable to the transmission of cholera, diarrhoea, skin diseases, COVID 19, malaria, cough, colds, Tuberculosis (TB), and pneumonia when staying at the GWS.

Patient guardians from public owned health facilities also highlighted safety, security, privacy, and well-being concerns. This was related to the location of the GWS, which were often far from the hospital. Rooms for sleeping were often congested (three people per square metre) (Fig 3) and had broken windows, poor lighting, no locks, and no means to secure their belongings in lockers.

“This GWS is isolated, with no fence nor electricity such that we are never safe as women” (Female FGD participant).

Staff responsible for delivering health talks to guardians reported that they were trained by the hospital management only on their roles and responsibilities regarding GWSs. Thus, they felt that they had limited technical knowledge on topics they were required to cover in health and hygiene talks. Instead, they used and applied their own knowledge acquired from other roles when delivering the health and hygiene talks at the GWS. Additionally, despite healthcare facility management reporting that the talks were delivered regularly, guardians reported that these talks were infrequent:

“The caretaker or chairperson call us at one place to communicate on a particular issue, but this only happens occasionally…. since I came here a week ago, we have been briefed on hygiene issues once” (IDI, female guardian).

### Ownership, and stakeholders responsible for conditions and management of the GWS

#### Ownership

There was significant variation in management and oversight of GWSs at government facilities (Table 3). Several stakeholders including the Members of Parliament, District Councils, Hospitals, and the wider community were identified as key stakeholders in the daily operation and maintenance and routine monitoring of the GWS. Interviews revealed a lack of clarity on who was responsible for GWS across multiple stakeholders. In several cases, respondents said that the GWSs were the responsibility of the hospital management since the GWSs were usually built on or near hospital grounds, and served people who were using the hospital. In other interviews, the hospital management and HAC members explained that the GWS belonged to the district council or the community.

#### Budget

Only GWSs serving private owned healthcare facilities (n = 2) had an allocated budget for maintenance of infrastructure and purchasing of necessary materials (i.e., cleaning materials). For public facilities, respondents reported using limited resources that were meant for patient wards, such as gloves and bleach for cleaning and disinfection to take care of the GWS.

“No. How can we budget for a thing which is not owned by us, it belongs to the district council. Sometimes as a hospital we just help because the GWS is near us” (IDI, Hospital management representative)

Thirty-three percent (n = 4) of GWSs (1 private and 3 public) reported collecting money from patient guardians (approximately USD 0.2 per patient guardian). GWS caretakers and chairpersons from GWS serving public hospitals indicated that the collection of fees was due to the lack of budget allocation for GWS while those from private owned hospitals indicated that sometimes the allocated budget is inadequate. The collected money was used for buying cleaning materials (e.g., hoes, brushes, brooms, and mops), and lighting materials (e.g., candles and torches). Collection of the fees was locally arranged by the GWS caretaker or GWS chairperson and collection frequency varied from upon arrival to only when the need arose.

There was mixed reception to payment among guardians. Some felt it was a good idea since this money was used to improve their wellbeing and overall GWS conditions, while others did not appreciate this approach as they already had financial challenges to take care of their patients and had the expectation that the hospital management should cover such costs for the GWSs.

### Stakeholders responsible for conditions and management

Forty-two percent (n = 5) of the GWSs had a paid caretaker (two from private and three from public facilities). The caretaker was responsible for cleaning the bathrooms, toilets, and the surrounding environment. At nine GWSs, a chairperson for each sleeping room was appointed from among the guardians staying in the facility (Table 3). The GWS chairperson was responsible for maintaining order at the GWS, solving disputes among patient guardians, allocating sleeping space to new guardians, and organising cleaning of the GWS. GWSs with a caretaker had generally better hygienic conditions than those without caretakers while there were no noticeable improvements in hygienic conditions among facilities with and without chairpersons. GWS chairpersons reported that they had limited authority as volunteers and other guardians did not respect them.

“It is difficult to maintain cleanliness here at the GWS because each time I try to organise my fellow patient guardians to clean the shelter, they always talk ill saying this is not my home, just mind your own business” (IDI, GWS Chairperson).

Respondents acknowledged that GWSs suffer from the lack of standards and policies to ensure there is a common strategy to design, deliver, operate, maintain, and monitor the facilities. This issue was further exacerbated by the absence of clarity of ownership and governance of GWSs at the district and national level.

‘‘We can’t develop standards for a thing which doesn’t belong to us. The first thing to be established is ownership for that facility, from there, issues of standards and monitoring can be resolved” (IDI, DEHO).

Some respondents highlighted the absence of an Infection Prevention and Control (IPC) policy targeting GWSs as “it is associated as being low risk” (IDI, DEHO) and beyond the scope of existing guidelines focusing on medical facilities. Meanwhile, one respondent challenged that an IPC policy should also focus on the GWSs with the following reason.

‘‘That area can be an important source of diseases. For example, people are sleeping on floors, so in terms of scabies, the diseases can spread very fast” (KII, DEHO).

Several HAC respondents raised concerns about tensions between HAC members and guardians. Guardians often disregarded requests from HAC members to take specific actions to improve conditions in GWSs (e.g., keeping latrines, cooking, and sleeping rooms clean and tidy). HAC members suggested this was linked to the lack of any visible identification–such as an identification (ID) or uniform–that signified their official role within the health system. Caretakers were also responsible for encouraging hygienic use and maintenance of GWS infrastructure.

### Findings from problem tree analysis

**Fig 7:**
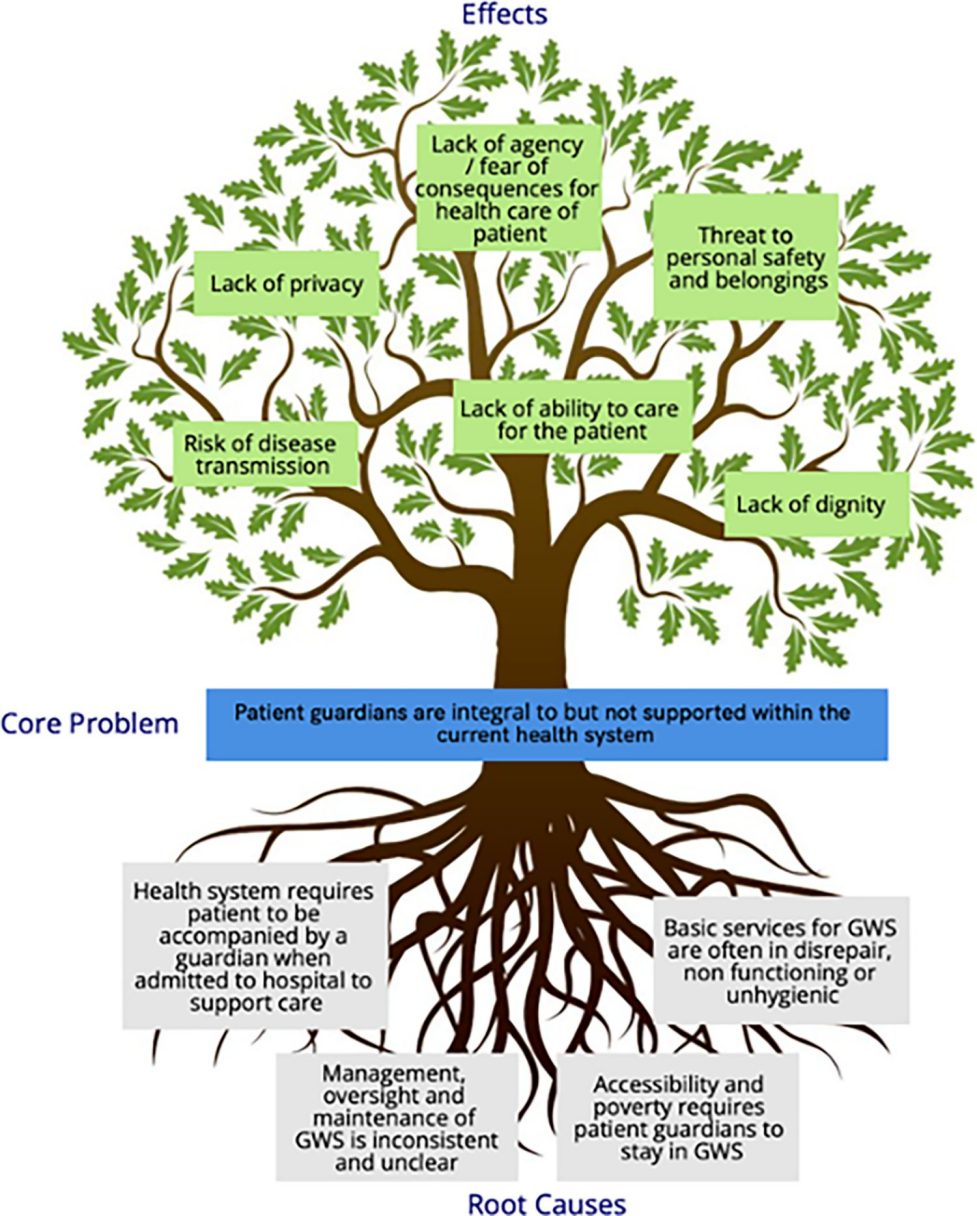
Problem tree analysis summarising findings of Guardian waiting shelter assessment.

Findings from both interviews and literature reinforced the fact that patient guardians are an integral yet neglected part of the health care system, including their ability to access and utilise the GWS to support their role. With this in mind, we used a problem tree analysis to triangulate our findings and separate the overarching problem from their cause-and-effect relationships. This enabled us to summarise the environmental, social, and economic challenges for patient guardians to effectively utilise GWSs in their current state (Fig 7).

Data collection tools and supporting data for this study are provided in the supplementary information (S1 Checklist, S2 Checklist data and S1 File and focus group discussion guide).

## Discussion

This study examined the GWS management structures, assessed available infrastructure and assessed guardian’s perception of health risk from GWS use. GWS, particularly those serving public hospitals in our study, have poor environmental health services and have limited infrastructure to provide for the comfort and safety of guardians. The study established that the GWSs are utilised by not only patient guardians but also serve as maternity waiting homes (MWHs) and are used daily by local vendors and members of the community with disabilities who have no formal support service. Our study highlighted lack of proper management structures for GWSs in Malawi as contributing to inadequate and poor infrastructure in GWSs thereby affecting the welfare of users.

The lack of clear ownership and accountability for the GWS was a consistent root cause of other challenges facing effective management and maintenance of the facilities for patient guardians. This was particularly pronounced at public health facilities, where the lack of clear ownership, budget, and accountability systems to ensure effective conditions and management, make it near impossible to manage access and maintain better standards for the patient guardians. Where improved systems were in place, such as employed caretakers or privately manged facilities, GWSs were able to maintain more hygienic and acceptable standards. Clarifying roles and responsibilities for management and maintenance of facilities has also been shown to be effective for the management of MWH where policies, management roles and finances are incorporated in the MWH model [32], and there is a clear recognition that health facilities are central to providing this support [33]. Placing GWSs under direct and clear hospital management would also offer a strategic opportunity to formalise existing supporting structures such as the HAC who already have an informal role in GWS operations and have been found to be an effective model for oversight and management of health facilities in other contexts [34]. Clearer management would also enable the role of GWS chairpersons to be re-evaluated, providing an opportunity to serve as a conduit for patient guardian communications and support the development of social capital in the group, rather than oversight of the facility for which they do not have the authority and are not compensated. More formal management would also enable clearer and realistic training for caretakers and chairpersons on technical subjects such as mitigating the risk of diarrhoeal and respiratory infections and providing them with appropriate acknowledgement of their role (e.g., ID, uniform).

There are currently no documented standards for patient guardian shelters in Malawi, or similar settings, which outline minimum infrastructural requirements for these settings. Although all GWSs in this study had access to safe water, some collected the water from outside the GWS premise which could compromise the water quality and quantity and limit adequate hygiene behaviours [35–37]. Lack of hygienic sanitation facilities also drove some guardians to practice open defaecation in the immediate area, further eroding sanitary conditions of the GWSs and introducing additional disease risks. This study highlights the complex repercussions of poor infrastructure and services in these settings. Not surprisingly, the risk of disease transmission, because of poor WASH facilities, is high not only between patient guardians within the GWS, but also between the hospital and the GWS residents as people move between wards and overcrowded, poorly ventilated and unclean accommodation [38]. However, it is also clear that the poor infrastructure and services have an additional impact on safety, security, dignity, and mental wellbeing of patient guardians. All of these reduce their ability to look after those in their care effectively, while addressing the additional stresses and burdens facing them around income and provision of support [2,39,40]. With this in mind, a minimum standard for GWS infrastructure and services should be developed, as has been undertaken for other areas of the health service [41,42]. The provision of these standards will support effective management and accountability, and provide the services needed for the third key area of integrating IPC.

As reported elsewhere, the presence of guidelines for managing MWH in Malawi have contributed to smooth running and improved IPC of such premises [41,42]. Therefore, the development and integration of specific guidelines for the GWSs would be beneficial in improving the welfare of patient guardians while staying in the GWS. Guidelines should seek to encompass the GWS into the whole system of the health facility, taking advantage of readily available resources, and incorporating already existing IPC policy and guidelines for Malawi [41–43] to provide consistency of approach and a sense of ownership. However, they must also take into consideration the education and literacy levels of caretakers, HAC members and patient guardians, and as such an illustrative and behaviour centred approach to guideline development is essential if they are to be effective. Guidelines should also include the regular provision of health promotion activities which are delivered by skilled and competent personnel.

The study established mixed levels of social capital among the patient guardians. For those who indicated to have good relations with other fellow patient guardians, they expressed a better experience while staying at the GWS with a sense of belonging and support. High levels of social capital in a community have been proven to promote participation of community members on various development activities and improves their coping mechanisms [22,44,45]. It is anticipated that if the standards and systems within the GWSs were to improve, this would lead to a similar improvement in social capital within the patient guardian population. With the observed limited resources at the GWSs, promoting social capital among patient guardians is vital in both improving and maintaining the conditions of the GWSs.

With this in mind, the study has identified three key areas which the Ministry of Health (MoH) can consider for further intervention: (1) Full integration of GWSs into the health care system; (2) Development of clear standards for GWSs encompassing minimum requirements for infrastructure, services, and security; and (3) Integration of GWS into hospital IPC systems. All three interventions would improve GWS users experience and ensure that minimum standards of facilities are defined and maintained. Additionally, improving the patient guardian experience could therefore enhance patient care, as well as reducing the risk of GWS acting as a vehicle for infection transmission.

## Study strengths and limitations

A key strength of this study was that our study was conducted in a neglected and yet an important component of the Malawi’s health care system. The study population was inclusive as we interviewed a range of key informants from the users to the managers of the GWSs.

It is important to consider certain constraints when analysing these findings. Some data from this study was based on self-reported data which is prone to bias as the participants may not recall all aspects of interest and may provide information based on what the researcher wants to hear. Where possible self-reported data has been validated through observation. GWSs in Malawi are present in both secondary and tertiary hospital settings; however, conducting the study in selected secondary level hospitals limits the generalizability of the present findings to other district and third tier hospitals in Malawi. Further, GWS management may have additional stakeholders at central government level to those recruited in this study; thus, future similar studies should consider targeting IPC and health facility infrastructure managers at Ministry of Health headquarters. Nevertheless, this study provides a better platform to understand the GWS context in Malawian hospitals for the subsequent design of interventions to improve the welfare of patient guardians.

## Conclusion and recommendations

GWSs remain integral to health care delivery in Malawi but are not effectively supported in current systems. The findings of this study call for the need for clear GWS management and ownership to facilitate improved welfare for the patient guardians, who plays an important role in health service delivery in Malawi. Such ownership should ensure that GWSs have dedicated budgets and staff. Importantly, much as specific guidelines for GWSs are essential, the need for the GWS management to be integrated into existing hospital IPC policy and guidelines cannot be overemphasized. Further, GWSs are potential touch points for health promotion among patient guardians and indirectly community members. Relatedly, future interventions aimed at improving patient guardian welfare in the GWSs should incorporate social capital strengthening. These study findings provide a basis from which a more detailed understanding of the GWS context in Malawi can be built.

## Supporting information

S1 Checklist GWS WASH CHECKLIST. (DOCX)

S2 Checklist (XLSX)

S1 Appendix Characteristics of the patient guardians (n = 221). (DOCX)

S1 File (DOCX)
