# Supplementary material for: Assessment of infrastructure, behaviours, and user satisfaction of guardian waiting shelters for secondary level hospitals in southern Malawi
Source: PLOS Glob Public Health. 2024 Jul 24;4(7):e0002642. doi: 10.1371/journal.pgph.0002642 (PMC11268604; doi:10.1371/journal.pgph.0002642)
Supplement: S1 Checklist — (DOCX) [file pgph.0002642.s001.docx]

GWS WASH CHECKLIST

| **Question** | **Responses** | **Code** |
| --- | --- | --- |
| Date & time of data collection: |  |  |
| Name of data collector: |  |  |
| Facility name: |  |  |
| District: |  |  |
|  |  |  |
| General information |  |  |
| How many sleeping rooms (in total) does the GWS have? | Insert number |  |
| How many sleeping rooms for females has the GWS have? | Insert number |  |
| How many sleeping rooms for males does the GWS have? | Insert number |  |
| What sleeping materials do the guardians use? | Mattresses |  |
|  | Mats |  |
|  | Rice bags |  |
|  | Beddings |  |
|  | Other….... (specify) | 77 |
| What is the source of the sleeping materials | Self-sourced | 1 |
|  | Provided by the hospital | 2 |
|  | Other …. (specify) | 77 |
| How many workers does the GWS have? | Insert number |  |
| For each of the cadres, how many are they? |  |  |
| Health surveillance assistant | Insert number |  |
| Guards | Insert number |  |
| Caretaker (Gardener) | Insert number |  |
| Cleaner | Insert number |  |
| Other…. (specify) | Insert number |  |
| Does the GWS have a specific kitchen/cooking area? | Yes | 1 |
|  | No | 0 |
| What cooking materials are used by the guardians? | Wood | 1 |
|  | Charcoal | 2 |
|  | Maize cobs | 3 |
|  | Other .... (specify) | 77 |
| Does the cooking area provide enough ventilation? | Yes | 1 |
|  | No | 2 |
| Water |  |  |
| Does the GWS have water? | Yes | 1 |
|  | No | 0 |
| If yes, what is the type of water source? | Public tap/standpipe | 1 |
|  | Borehole | 2 |
|  | Protected dug well | 3 |
|  | Unprotected dug well | 4 |
|  | Protected spring | 5 |
|  | Unprotected spring | 6 |
|  | River/stream | 7 |
|  | Drainage water | 8 |
|  | Rainwater collection | 9 |
|  | Other...(specify) | 77 |
| How are the water bills settled? | Prepaid | 1 |
|  | Post paid | 2 |
|  | Free | 3 |
| Is the improved water supply piped into the facility or located on premises? | On premises | 1 |
|  | Within 500m | 2 |
|  | Further than 500m | 3 |
|  | Not applicable | 99 |
| Is water available at the time the assessment is carried out? | Water is available throughout the facility | 1 |
|  | Water is available from some but not all water points | 2 |
|  | No water is available | 3 |
| If water is available, check the colour of the water | Clear with no particles | 1 |
|  | Clear with a few particles | 2 |
|  | Turbid | 3 |
|  | Other...(specify) | 77 |
| Has main water supply system been functional for the last 2 weeks | Yes | 1 |
|  | No | 0 |
| Why has the water supply system been non-functioning? | Water supply system has breakdown within the vicinity of the GWS | 1 |
|  | Water supply system has breakdown outside the vicinity of the GWS | 2 |
|  | Water bill has not been sorted/ water units not bought | 3 |
|  | Other...(specify) |  |
| Does the facility have additional (alternative) improved water source(s) available | Additional improved water source identified, available and sufficient | 1 |
|  | Additional water source identified but not improved or not sufficient | 2 |
|  | No additional water source available | 1 |
| Additional improved water source identified, available and sufficient, do they have to pay to access or fetch the water? | Free of charge | 1 |
|  | Paying | 2 |
| If additional water source identified is not improved or not sufficient, what is the type of the alternative source | Unprotected dug well | 1 |
|  | Unprotected spring | 2 |
|  | River/stream | 3 |
|  | Drainage water | 4 |
|  | Other...(specify) | 77 |
| How far is the alternative source of water from the market? | Collection time of not more than 30 minutes of round-trip including queuing | 1 |
|  | Collection time exceeds over 30 minutes of round-trip including queuing | 2 |
| Is there a specific source for drinking water? | Yes | 1 |
|  | No | 0 |
| If yes, what is the source of the drinking water? | Public tap/standpipe | 1 |
|  | Borehole | 2 |
|  | Protected dug well | 3 |
|  | Unprotected dug well | 4 |
|  | Protected spring | 5 |
|  | Unprotected spring | 6 |
|  | River/stream | 7 |
|  | Rainwater collection | 8 |
|  | Other...(specify) | 77 |
| Sanitation |  |  |
| Does the GWS have toilets? | Yes | 1 |
|  | No | 0 |
| If yes, are they improved toilets? NB: Improved sanitation facilities: include flush toilets into managed sewer or septic tank and soakaway pit, VIP latrines, pit latrines with slab and composting toilets. | Yes | 1 |
|  | No | 0 |
| How many improved toilets does the GWS have? | Insert number |  |
| Toilets are clearly separated/labelled for male and female or provide privacy (i.e. single stall/room) | Separate toilets for male/female use exist and are clearly labelled (and provide privacy for users) | 1 |
|  | Separate toilets exist but not clearly labelled | 2 |
|  | No separate toilets exist or no privacy in gender neutral toilets | 3 |
| Are all available toilets usable? (usable means, accessible, functional,  and private) | All toilets available are usable | 1 |
|  | Some toilets available are usable | 2 |
|  | None of the toilets are usable | 3 |
| Number of usable toilets in total | Insert number |  |
| How many are of the usable toilets for men | Insert number |  |
| How many are of the usable toilets for women | Insert number |  |
| Does the GWS have special toilets for staff? | Yes | 1 |
|  | No | 0 |
| Does other people use the toilets (e.g. Vendors) | Yes | 1 |
|  | No | 0 |
| Assessment of each usable toilet |  |  |
| Type of toilet available | Pour flush | 1 |
|  | Traditional pit latrine | 2 |
|  | Improved pit latrine (with slab) | 3 |
|  | Ventilated improved latrine | 4 |
|  | Ecosan latrine | 5 |
| Who are the users of the toilets? | Male | 1 |
|  | Female | 2 |
| Does the latrine provide privacy (privacy means the latrine has a door or E shaped wall) | Yes | 1 |
|  | No | 2 |
| Observe. Check presence of faeces on latrine walls | Yes | 1 |
|  | No | 2 |
| Can the toilet/latrine be easily used by the children? | Yes | 1 |
|  | No | 0 |
| Can the toilet/latrine be easily used by the disabled? | Yes | 1 |
|  | No | 0 |
| Observe. Presence of anal cleansing materials in the latrine | yes | 1 |
|  | No | 2 |
| If yes, what material is present (multiple responses) | Toilet paper | 1 |
|  | Newspaper/ cardboard | 2 |
|  | Leaves | 3 |
|  | Stones | 4 |
|  | Water | 5 |
| Check presence of handwashing facility near latrine | Yes | 1 |
|  | No | 2 |
| If yes, is the handwashing facility user friendly among the children and the disabled? | Handwashing facility can be easily used by the disabled | 1 |
|  | Handwashing facility can be easily used by children | 2 |
|  | Both children and the disabled can easily use the handwashing facility | 3 |
|  | Children or the disabled cannot easily use the handwashing facility | 4 |
| Type of handwashing facility within latrine premise | Standpipe | 1 |
|  | Tippy tap | 2 |
|  | Bucket with tap | 3 |
|  | Basin and jug | 4 |
|  | Basin only | 5 |
| Check presence of handwashing materials | Soap and water | 1 |
|  | water only | 2 |
|  | Ash and water | 3 |
|  | None is available | 4 |
| Is handwashing wastewater collected after use? (Drainage) | Yes | 1 |
|  | No | 2 |
| How is the wastewater collected? | Drainage system | 1 |
|  | Bucket | 2 |
|  | Other...(specify) | 77 |
| Does the toilet provide the means to manage menstrual hygiene management (MHM) needs | Yes | 1 |
|  | No | 2 |
|  | Not applicable | 99 |
| Hand Hygiene |  |  |
| Functioning hand hygiene stations are available in the guardian waiting yard (other than handwashing facilities at the toilets) | Yes | 1 |
|  | No | 0 |
| Insert number of handwashing facilities present | Insert number |  |
| Assessment of each handwashing facility |  |  |
| Where is the handwashing facility located? | At the entrance of the GWS | 1 |
|  | In the GWS yard | 2 |
|  | Within the cooking area | 3 |
|  | Other...(specify) | 77 |
| Type of handwashing facility present | Standpipe | 1 |
|  | Tippy tap | 2 |
|  | Bucket with tap | 3 |
|  | Basin and jug | 4 |
|  | Basin only | 5 |
| Check presence of handwashing materials | Soap and water | 1 |
|  | water only | 2 |
|  | Ash and water | 3 |
|  | None is available | 4 |
| Check presence of water at the handwashing facility | Yes | 1 |
|  | No | 0 |
| If bucket with tap, what is the position of the handwashing facility | Reasonable height | 1 |
|  | Too low | 2 |
|  | Too high | 3 |
| How is the wastewater collected? | Drainage system | 1 |
|  | Bucket | 2 |
|  | Other...(specify) | 77 |
| Is the handwashing facility user friendly among the children and the disabled? | Handwashing facility can be easily used by the disabled | 1 |
|  | Handwashing facility can be easily used by children | 2 |
|  | Both children and the disabled can easily use the handwashing facility | 3 |
|  | Children or the disabled cannot easily use the handwashing facility | 4 |
| Hand hygiene promotion materials are displayed and clearly visible within the guardian waiting shelter | Yes | 1 |
|  | No | 0 |
| If yes, what message do they convey? | Key times for handwashing | 1 |
|  | Handwashing technique | 2 |
|  | Safe water chain practices | 3 |
|  | Clean use of latrines | 4 |
|  | Responsible disposal of solid waste | 5 |
|  | Physical distancing | 6 |
|  | Mask wearing | 7 |
|  | General COVID-19 preventive measures | 8 |
|  | Other...(specify) | 77 |
| Security |  |  |
| Is the facility fenced? | Yes | 1 |
|  | No | 0 |
| If yes, what is the type of the fence? | Wire fence | 1 |
|  | Brick fence | 2 |
|  | Grass or bamboo fence | 3 |
|  | Other...(specify) | 66 |
| Does the facility have electricity for lighting? | Yes | 1 |
|  | No | 0 |
| Waste Management |  |  |
| Functional waste collection containers are available near all waste generation points e.g. cooking area | Functional waste collection containers exist at all waste generation points | 1 |
|  | Functional bins at some but not all waste generation points | 2 |
|  | No bins or separate waste disposal | 3 |
| Is waste segregated at point of generation? | Yes | 1 |
|  | No | 2 |
| Appropriate protective equipment and resources to perform hand hygiene are available for all staff responsible for handling waste and in charge of waste treatment and disposal | Resources for hand hygiene and protective equipment available | 1 |
|  | Some equipment available, but not for all staff, or available but damaged | 2 |
|  | No equipment available for staff | 3 |
| A dedicated waste disposal area is available which is fenced and secure | Dedicated and fenced waste disposal area available, of sufficient capacity | 1 |
|  | Dedicated waste disposal area available but not fenced or secure or not sufficient capacity | 2 |
|  | No dedicated waste disposal area available | 3 |
| What is the condition of the waste disposal site | Waste disposal site too full | 1 |
|  | Waste disposal site not full | 2 |
| Is the waste disposed properly into the waste disposal area (pit) NB: To check indiscriminate disposal of waste around the disposal area | Yes | 1 |
|  | No | 2 |
| How Is waste treated after disposal | Burned | 1 |
|  | Landfilling | 2 |
|  | No treatment | 3 |
|  | Other...(specify) | 77 |
