# Supplementary material for: Assessment of infrastructure, behaviours, and user satisfaction of guardian waiting shelters for secondary level hospitals in southern Malawi
Source: PLOS Glob Public Health. 2024 Jul 24;4(7):e0002642. doi: 10.1371/journal.pgph.0002642 (PMC11268604; doi:10.1371/journal.pgph.0002642)
Supplement: S1 Appendix — (DOCX) [file pgph.0002642.s003.docx]

APPENDIX 1**: Characteristics of the patient guardians (n=221)**

| **Variable** | **Percentage (n=221)** |
| --- | --- |
| **Guardian’s relationship to patient** | |
| Brother | 13.55 |
| Daughter | 39.25 |
| Daughter in law | 4.21 |
| Mother | 3.74 |
| Sister | 13.08 |
| Son | 9.35 |
| Others (Wife, Grandchild, sister-in-law, mother-in-law, uncle, and father) | 16.82 |
| **Level of education** | |
| Never attended | 23.53 |
| Primary | 62.44 |
| Secondary | 13.12 |
| Tertiary | 0.91 |
