# Supplementary material for: Assessment of infrastructure, behaviours, and user satisfaction of guardian waiting shelters for secondary level hospitals in southern Malawi
Source: PLOS Glob Public Health. 2024 Jul 24;4(7):e0002642. doi: 10.1371/journal.pgph.0002642 (PMC11268604; doi:10.1371/journal.pgph.0002642)
Supplement: S1 File — (DOCX) [file pgph.0002642.s004.docx]

**Key Informant guide Hospital management representative**

| Date |  | Time |  |
| --- | --- | --- | --- |
| District Hospital Name |  | Enumerator Name |  |

***IDI Details***

| Gender |  | | |
| --- | --- | --- | --- |
| Age |  | Profession | Nurse / Doctor / Midwife / Cleaner / GWS Chair / Guardian / Other |
| Time spent in present District Hospital / GWS? |  | Time spent in current position |  |

***District Level Hospital Services & Demographics***

| Which of the following services or departments are available at this healthcare facility? | - Emergency Department - Immunization - Outpatient - Inpatient - Intensive Care Unit - Pharmacy - Labour and delivery - Postnatal - Antenatal care - Nutritional services - Minor surgery - Major surgery - Other | |
| --- | --- | --- |
| How many inpatient beds are available at the HCF? |  | |
| On an average day, how many inpatients are admitted? |  | |
| On an average day, how many outpatients are seen? |  | |
| How many non-medical staff are employed at the healthcare facility? |  | |
| How many medical staff are employed at the healthcare facility? |  | |
| As of today, can you please describe the typical health issues which are referred to your healthcare facility?  Probe for any trends across age, gender, seasons, urban/rural areas etc. |  | |
| Given the unprecedented COVID-19 global pandemic can you describe your immediate and long-term response plan?  Probe on issues such as the continuation of preventative behaviours, infection versus hospitalisation, variants, long COVID-19, misinformation, vaccination etc. |  | |
| Does this facility have linkages with providers of traditional, complementary or other integrative types of medicine? | Yes | No |
| Can you please describe these linkages and how they are implemented? |  | |
| Does the facility support/manage community health workers (CHWs)? | Yes | No |
| For which services are the CHWs active in the community? | - HIV - TB - Malaria - Diarrhoeal diseases - Maternal health - Follow up of patients with chronic illnesses - Nutritional concerns - Vaccination - Growth monitoring - Other | |
| How do the CHWs engage with the community? | - House to house/face-2-face - Hygiene sessions during mobile clinics - Hygiene sessions during immunization campaigns - Hygiene sessions during distributions - Hygiene sessions at schools - Hygiene sessions at other public places – specify ………………… - SMS - Interactive Voice Recordings - Broadcasts via radio and/or TV - Other | |

Our focus is the Guardian Waiting Shelters, and we would welcome your perspectives in the following –

***Essential Components - GWS***

| What is of critical importance to the functionality and use of the GWS?  *[Open question]*  Prompt research respondents to highlight up to five areas and enumerators should select from the following categories - | - Hand hygiene - Food hygiene - Water quality - Water quantity - Latrine use - Vector control - Non-medical waste management - Medical waste management - Gender sensitivity - Privacy - Dignity - Security - Community engagement - Exchange of goods & services - Other |
| --- | --- |
| Now ask the respondent to elaborate on the infrastructure, services, good, behaviours, which are essential at the GWS? | Why it is important  Risks  How they are mitigated |
| Today, what successes do you have in providing WASH and IPC infrastructure/services/goods at the GWS? |  |
| Today, what specific challenges do you have in providing WASH and IPC infrastructure/services/goods at the GWS? |  |

***Perceptions***

| What is the perceived role of the GWS in accessing the healthcare assistance at the District Hospital?   1. How is this role perceived by you? 2. How is this role perceived by staff? 3. How is this role perceived by guardians? 4. How is this role perceived by the wider community? |  | |
| --- | --- | --- |
| Is it common practice to inform Guardians about the importance of WASH/IPC behaviours at your GWS facility? | Yes | No |
| If yes, please specify – how (method), what (topic), who (which personnel conducts them), when (frequency, and time of day) |  | |
| How do you consider the effort required by Guardians to perform good WASH/IPC behaviours when caring for patients at your GWS facility? | No effort - - - - - - - - - - - - - - - - - - - - - - - -A big effort | |
| How do Guardians perceive your request to perform optimal WASH/IPC behaviours during patient care at your GWS facility?  *impt = importance | Of little impt - - - - - - - - - - - - - - - - - - - - - - Very impt | |

***Monitoring and Implementation of Systems for Quality Assurance***

| Does this healthcare facility participate in any periodic assessment of conditions in the GWS against standards, where a resulting score or status is provided? | Yes | No |
| --- | --- | --- |
| What metrics/standards do you use? |  | |
| When was this last carried out? |  | |
| What were the findings? |  | |
| How frequent are these checks and balances carried out? |  | |
| If nothing is carried out specific to the GWS – please repeat these questions for the healthcare facility (this will inform readiness to be adopted to the GWS) |  | |

***Training & Education***

| Does this healthcare facility provide training to medical staff on WASH & IPC? | Yes | | | | | | | | No | | | | | | | |
| --- | --- | --- | --- | --- | --- | --- | --- | --- | --- | --- | --- | --- | --- | --- | --- | --- |
| How often do these pieces of training occur? | Monthly | | Quarterly | | | | Every 6 months | | | | | Annually | | | Other | |
| Does this healthcare facility provide training to non-medical staff (not involved in direct patient care) on WASH and IPC? | Yes | | | | | | | | No | | | | | | | |
| How often do these pieces of training occur? | Monthly | Quarterly | | | | Every 6 months | | | | | Annually | | | Other | | |
| Are any of the trained personnel responsible for the WASH and IPC measures at the GWS? | Yes | | | | | | | No | | | | | | | | |
| If yes, can you please specify their position and typical duties at the GWS? |  | | | | | | | | | | | | | | | |
| Are campaigns/sessions conducted at the GWS, which target behaviours of the Guardians? | Yes | | | | | | | No | | | | | | | | |
| If yes, how often | Daily | | | Weekly | Monthly | | | | | Quarterly | | | Every 6 months | | | Other |
| Please can you elaborate more on the campaigns/sessions   - Target audience - Targeted message(s) - Methodology - Who conducts the sessions - Effectiveness - Etc. |  | | | | | | | | | | | | | | | |

***Funding & Procurement***

| Do you have an annual budget for the healthcare facility that included funding for WASH & IPC infrastructure, services, personnel and the continuous procurement of WASH & IPC items? | Yes | No |
| --- | --- | --- |
| What % of your monthly budget is spent on WASH and IPC provisions specific to the GWS? | % | |
|  | Major GWS WASH & IPC expenditures include – specify items | |
|  | Minor GWS & IPC expenditures include – specify items | |
| Is there a central purchasing unit for the entire healthcare facility? | Yes | No |
| Does this include the GWS? | Yes | No |
| Please describe the process of purchase and distribution of products (WASH and IPC in nature) for the GWS?   - How often - Staff responsible - Items distributed - Process - Etc. |  | |
| Would you like to communicate anything else about funding and distribution across the different departments – successes and challenges? |  | |

***Other research***

| Are you aware of other research – past/present/future conducted, which has had a focus on GWS? |  |
| --- | --- |
| Are you aware of other research – past / present / future conducted, which has had a focus on maternity waiting wards? |  |

***AOB***

| Is there anything else you would like to communicate to use, which would support our research? |  |
| --- | --- |

We would like to thank you for your participation and look forward to sharing the outcome of our findings.

**Interview guide for Health Advisory Committee and caretaker**

| Date |  | Time |  |
| --- | --- | --- | --- |
| District Hospital Name |  | Enumerator Name |  |

***IDI Details***

| Name of Research Respondent |  | Gender |  |
| --- | --- | --- | --- |
| Age |  | Profession | Caretaker / HAC Representative |
| Time spent in present District Hospital / GWS? |  | Time spent in current position |  |

***Roles at the GWS***

| Can you tell me about your role in the functionality and use of the GWS? |  | |
| --- | --- | --- |
| What is the perceived role of the GWS in accessing healthcare assistance at the District Hospital?   1. How is this role perceived by you? 2. How is this role perceived by guardians? 3. How is this role perceived by the wider community? |  | |
| Is it common practice to inform Guardians about the importance of WASH/IPC behaviours at your GWS facility? | Yes | No |
| If yes, please specify – how (method), what (topic), who (which personnel conducts them), when (frequency, and time of day) |  | |
| How do you consider the effort required by Guardians to perform good WASH/IPC behaviours when caring for patients at your GWS facility? | No effort - - - - - - - - - - - - - - - - - - - - - - - - - - - - - - - -A big effort | |
| How do Guardians perceive your request to perform optimal WASH/IPC behaviours during patient care at your GWS facility?  *impt = importance | Of little impt - - - - - - - - - - - - - - - - - - - - - - - - - - - - - - Very impt | |

***Hazards/problems and risk assessment***

| What do you think are the main problems and risks associated with WASH & IPC at the GWS? | | | |
| --- | --- | --- | --- |
| Area | Hazards/problems identified | Risks | How to mitigate them |
| Water |  |  |  |
| Sanitation |  |  |  |
| Hygiene |  |  |  |
| IPC |  |  |  |
| Management |  |  |  |

| In summary, what do you think are the successes in providing WASH & IPC infrastructure/services/goods at the GWS? |  |
| --- | --- |
| In summary, what do you think are the challenges in providing WASH and IPC infrastructure/services/goods at the GWS? |  |

***Training & Education***

| Did you receive formal training in WASH/hygiene behaviours in the last 2 years? |  |
| --- | --- |
| If yes, please describe  By whom  When and duration  Content  Key takeaways |  |
| Did you receive formal training in IPC in the last 2 years? |  |
| If yes, please describe -  By whom  When and duration  Content  Key takeaways |  |

***WASH & IPC measures***

| GWS has a functional quality management/ quality improvement / IPC or WASH FIT team, with designated focal persons from IPC, WASH, etc. | Team(s) exists, has clear TORs, meets regularly with good leadership and decisions are noted and followed up on  Team(s) meets but irregularly, informally, does not have clear TORs etc.  No such team(s) exist  Other |
| --- | --- |
| Water availability | Water is available 7 days a week, all day, every day  Water is available 5 days/week and/or not the entire day  Water is available fewer than five days per week and/or is not available for more than half the day  Other |
| Water is of sufficient quantity for all uses | Water is of sufficient quantity for all uses across the whole facility  Water quantity is sufficient for 75% of needs (across all wards and uses)  Water quantity less than 75% sufficient  Other |
| Toilet cleaning schedule - toilets are cleaned at least once each day and a record of cleaning is signed by the cleaners and displayed visibly? | Toilets cleaned each day and a signed record is visible 1  Toilets cleaned but less than once a day with or without record  No record available and toilets cleaned less than once a day 3  Other |
| Who is responsible for the cleaning of the toilets? | Guardians?  Guardian Chair?  GWS Caretake?  GWS Cleaner?  Other |
| Adequate PPE is available at all times and in sufficient quantities for all cleaning staff | All members of cleaning staff have adequate PPE  Some but not all staff have full PPE or PPE available but in poor condition  Not available  Other |
| A routine budget for environmental cleaning supplies and equipment exists and is sufficient for all needs. | Budget exists and is sufficient for all needs  Budget exists but is not sufficient for all needs  No budget exists  Other |
| Appropriate and well-maintained materials for cleaning (i.e., detergent, mops, buckets, etc.) are available and sufficient | All necessary equipment available, in good condition and sufficient  Available but not well maintained or in some but not all areas or not sufficient  No materials available  Other |
| Are regular audits of the GWS undertaken to assess the availability of hand sanitiser, soap, single-use towels and other hand hygiene resources? | Yes  No |
| How often? | Daily  Weekly  Monthly  Bi-monthly  Quarterly  Every 6 months  Other |
| Are audits on water quality periodically performed at the GWS? |  |
| Water quality – if yes, what is measured | Free residual chlorine  CFU  Turbidity  Other |
| Are audits on functionality and use of latrines carried out at the GWS? |  |
| How often? | Twice per day  Daily  Every 2^nd^ day  Weekly  Monthly  Other |
| Are audits on waste management performed at the GWS? |  |
| How often? | Twice per day  Daily  Every 2^nd^ day  Weekly  Monthly  Other |
| What feedback mechanisms are in place for healthcare staff, guardians, patients, community members should they wish to communicate something about the WASH & IPC conditions at the GWS? |  |
| What are the common themes raised? |  |

***AOB***

| Is there anything else you would like to communicate to use, which would support our research? |  |
| --- | --- |

**Focus group discussion guide for guardians**

**Location Details**

| Date |  | Start Time  End Time |  |
| --- | --- | --- | --- |
| District Hospital Name |  | District |  |
| FGD Facilitator |  | FGD Recorder |  |
| No of FGD Participants  (segregated by gender) |  | Range of locations where guardians have come from – which Districts |  |

| Question | What participants said | Analysis – key takeaways |
| --- | --- | --- |
| Can you describe your typical family life back in your community, with a specific to access to water and sanitation and practice of hygiene behaviours? |  |  |
| Can you describe your typical role as a guardian at this guardian waiting shelter?  *Probe –* what duties do they do during the day, and during the night? |  |  |
| What is your perceived role of the GWS in health care utilisation? |  |  |
| What support do you get from your family, healthcare facility, GWS, and/or community to fulfil your role as a guardian? |  |  |
| Based on your own experience can you describe the functionality and use of the following GWS infrastructure?   1. Water supply 2. Latrines 3. Bathing 4. Handwashing 5. Food preparation and cooking area 6. Sleeping area 7. Other   *Probe ­*– during the discussion the successes and challenges |  |  |
| Which hygiene behaviours are you able to practice routinely at the GWS?  *Probe –* what has enabled them to do so – knowledge, motivation, fear, services, infrastructure, goods etc…. |  |  |
| Which hygiene behaviours are you not able to practice routinely at the GWS?  *Probe* – what has prevented them to do so |  |  |
| Have either of you attended education/awareness session whilst at the GWS?  If yes, please describe?  If no, do you have any ideas – topics, delivery methods ? |  |  |
| What do you like about the GWS? |  |  |
| What do you dislike about the GWS? |  |  |
| How could the GWS be improved? |  |  |
| What communication channels do you use whilst at the GWS to provide feedback?  If any participant has done so - ask them to explain more – the issue, who they fed back to, the outcome etc. |  |  |
| Is there anything else you would like to communicate which would support our understanding of your role as guardian and the |  |  |
